# Anomalous Right Coronary Artery Clinical Presentations and Considerations

**DOI:** 10.7759/cureus.57207

**Published:** 2024-03-29

**Authors:** Elizabeth L Allison, Kai Shiang Lin, Inna Bukharovich

**Affiliations:** 1 Internal Medicine, State University of New York Downstate Medical Center, Brooklyn, USA; 2 Cardiology, Kings County Hospital Center, Brooklyn, USA

**Keywords:** anomalous origin of right coronary artery, coronary computed tomography angiogram, cariogenic shock, unexplained syncope, nonischemic cardiomyopathy, n terminal pro bnp, decompensated heart failure, heart failure with reduced ejection fraction, "anomalous coronary artery"

## Abstract

Anomalous coronary artery presenting as syncope or acute decompensated heart failure complicated by cardiogenic shock is a relatively rare finding. Here, two unusual presentations are described in which an anomalous right coronary artery (RCA) with interarterial course was found following an initially negative workup. The first case describes a 71-year-old male with known non-ischemic cardiomyopathy presenting with acute decompensated heart failure and cardiogenic shock. The second case highlights a 44-year-old female presenting with intermittent angina and recurrent syncope of unknown etiology. These two cases suggest that the anatomy of coronary arteries and their anatomical variants may play a crucial role in the development of adverse cardiovascular outcomes. Utilizing cardiac computed tomography angiography with a lower threshold in patients presenting with cardiac signs, symptoms, and risk factors would lead to earlier detection of these anatomic anomalies and intervention either medically or surgically for potentially improved long-term outcomes.

## Introduction

Coronary artery anomalies are a diverse group of congenital disorders in which abnormal coronary embryogenesis results in abnormalities in the origin of coronary arteries [1]. These developmental rarities occur in less than 1% of the population and are characterized by numerous variations in their shape, origins, and location of ostia [2]. Despite its variable nature, an anomalous coronary artery is usually asymptomatic, although it can also present with clinically significant features such as acute decompensated heart failure, cardiogenic shock, syncope, and sudden cardiac death [3-9]. Clinical heterogeneity is a hallmark, and ominous presentations are often associated with clinically significant lesions such as the right coronary artery (RCA) originating from the left sinus of Valsalva or the left anterior descending artery (LAD) [10]. In this report, we present two unusual cases of anomalous RCA and highlight their unique clinical presentations, imaging findings, management implications, and prognostic importance.

## Case presentation

Case 1

**Figure 1:**
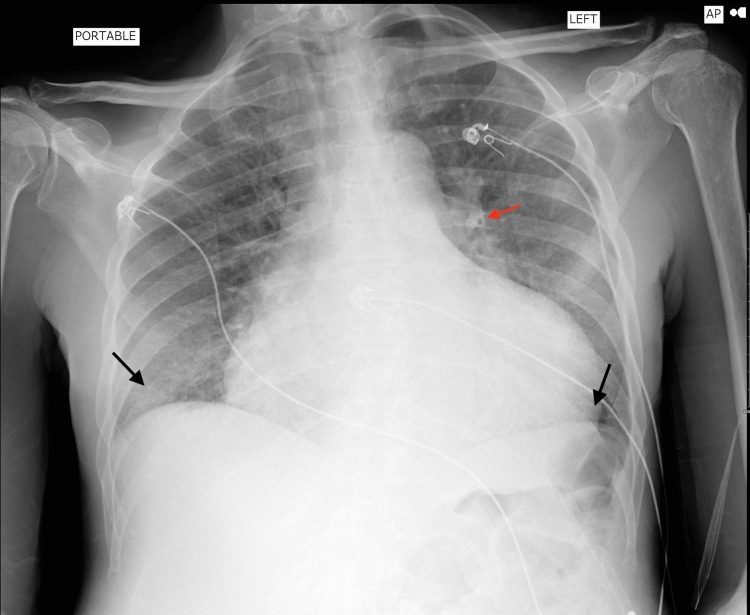
Anteriorposterior (AP) Chest X-ray: Black arrows point to regions of small bilateral pleural effusions without consolidations and the red arrow points to pulmonary vascular congestion.

**Figure 2:**
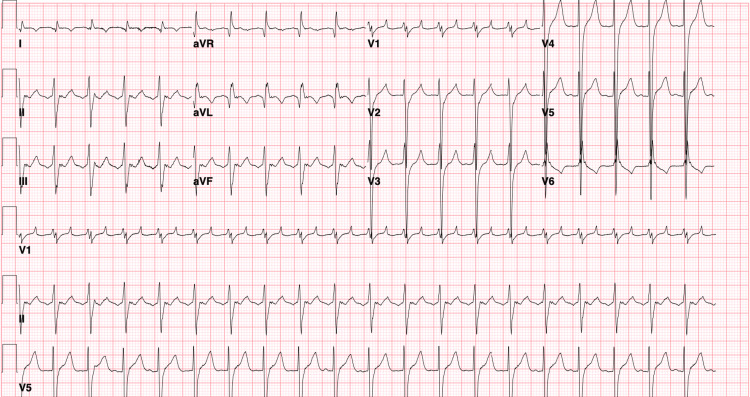
Electrocardiogram (EKG) showing atrial flutter with a 2:1 atrioventricular (AV) block

A 71-year-old male with a history of heart failure with reduced ejection fraction (HFrEF) of unknown etiology with a left ventricular ejection fraction (LVEF) of 5-10% and a recent admission for acute decompensated heart failure presents to the emergency room with worsening exertional and non-exertional dyspnea, orthopnea, and bilateral lower extremity edema. N-terminal pro‐B‐type natriuretic peptide (NT-proBNP) was found to be 11,492 pg/mL (Table 1), and a chest X-ray revealed pulmonary vascular congestion and small bilateral pleural effusions without consolidations (Figure 1). An electrocardiogram (EKG) showed atrial flutter and a 2:1 atrioventricular (AV) block (Figure 2). The patient was admitted to telemetry for management of acute decompensated heart failure.

**Table 1:** Laboratory values on admission

Test name	Patient 1	Patient 2	Reference Range	Units
Hemoglobin	13.3	13.0	14.0 – 18.0	g/dL
White blood cell count	6.17	5.11	4.50 – 10.90	K/uL
Platelets	291	268	130 – 400	K/uL
Troponin T	0.044	0.010	≤ 0.010	ng/mL
PRO B-Type Natriuretic Peptide	11,492	24	≤ 125	pg/mL
Blood Urea Nitrogen	39.0	13.0	8.0 – 23.0	mg/dL
Creatinine	1.73	0.77	0.70 – 1.20	mg/dL

**Figure 3:**
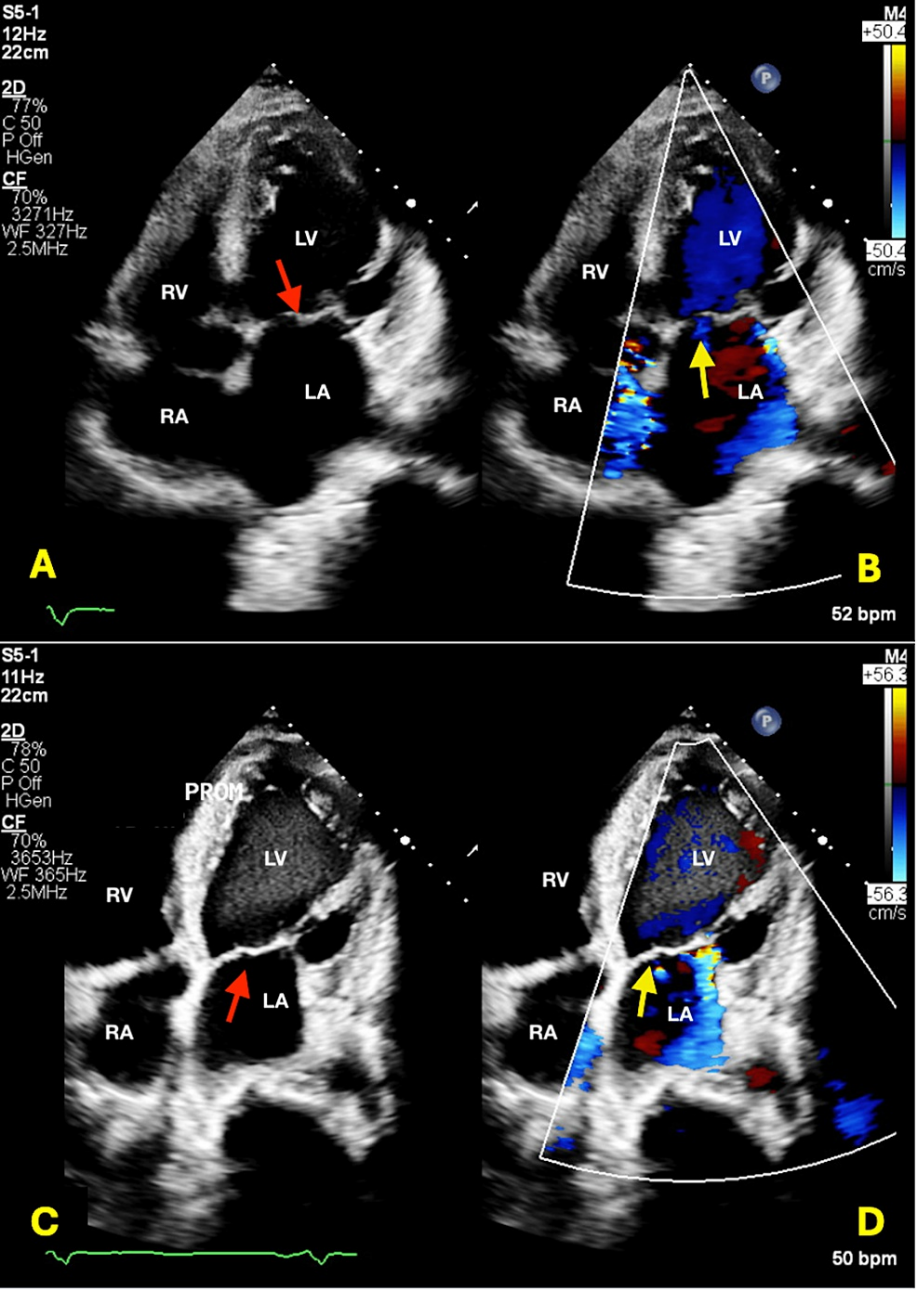
2D Transthoracic Echocardiogram: A & B: Akinetic heart, severe mitral regurgitation, ejection fraction of 25%; C & D: following mitral clip placement. Red arrows indicate valve pre and post mitral clip placement. Yellow arrows point to mitral valve with doppler flow. (B) shows mitral regurgitation and (D) indicates flow following mitral clip placement. RA: right atrium, RV: right ventricle, LA: left atrium, LV: left ventricle

**Figure 4:**
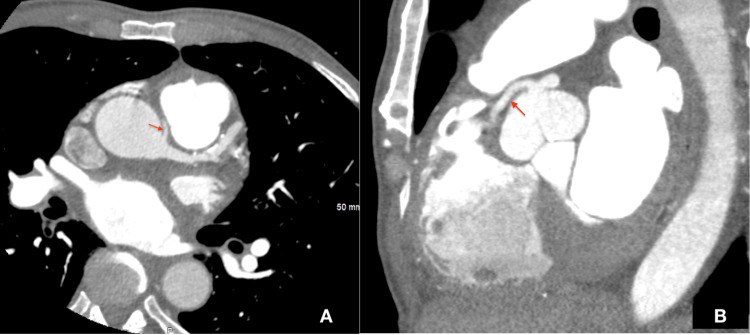
A: Cardiac computed tomography angiography (CCTA) showing a single coronary artery arising from the left coronary cusp. Red arrow indicates anomalous RCA angiography. B: CCTA demonstrating a single coronary artery arising from the left coronary cusp bifurcating into the RCA and LMCA. The arrow shows the anomalous RCA with an acute takeoff angle (approximately 17 degrees) taking an interarterial course between the aorta and the main pulmonary artery. RCA: right coronary artery, LMCA: left main coronary artery,

**Figure 5:**
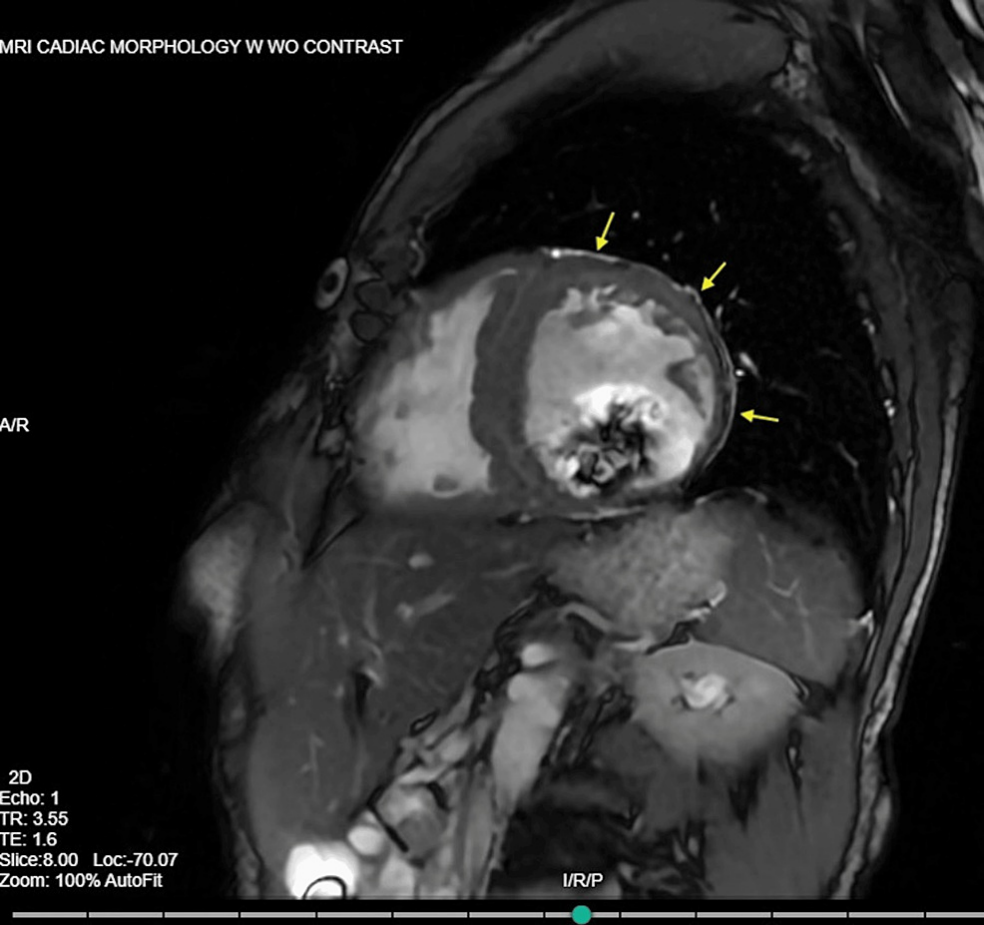
Cardiac Magnetic Resonance Imaging (MRI) showed moderately dilated ventricles and late gadolinium enhancement of the left ventricular free wall indicated by arrows with septal sparing.

His hospital course was complicated by cardiogenic shock requiring vasopressor support, inotropic support, and intra-aortic balloon pump (IABP) placement. A transthoracic echocardiogram (TTE) showed an akinetic heart, ejection fraction of 25%, and severe mitral regurgitation (MR) (Figure 3). Cardiac computed tomography angiography (CCTA) revealed a single coronary artery arising from the left coronary cusp (Figure 4A) giving rise to the RCA and the left main coronary artery (LMCA). With an acute angle takeoff, the RCA courses inter-arterially between the aorta and the main pulmonary artery (Figure 4B). Cardiac Magnetic Resonance Imaging (MRI) showed moderately dilated ventricles and late gadolinium enhancement of the left ventricular free wall with septal sparing (Figure 5).

The patient eventually underwent transcatheter edge-to-edge repair (TEER) of the mitral valve (Figure 3) and implantable cardiac defibrillator (ICD) placement for severe MR and sustained monomorphic ventricular tachycardia, respectively, and was discharged on guideline-directed medical therapy.

Case 2

A 44-year-old female with a history of hyperlipidemia, obesity, pre-diabetes, and an unknown seizure disorder presents to the office for a follow-up of intermittent chest pain associated with dizziness and shortness of breath that occurs at least once weekly. Additionally, she endorsed two episodes of syncope over the last several years not associated with exertional activity. The patient denied palpitations during either event and did not have orthopnea, paroxysmal nocturnal dyspnea, or cough at baseline, although she endorsed exertional dyspnea and had an exercise tolerance of two flights of stairs.

**Figure 6:**
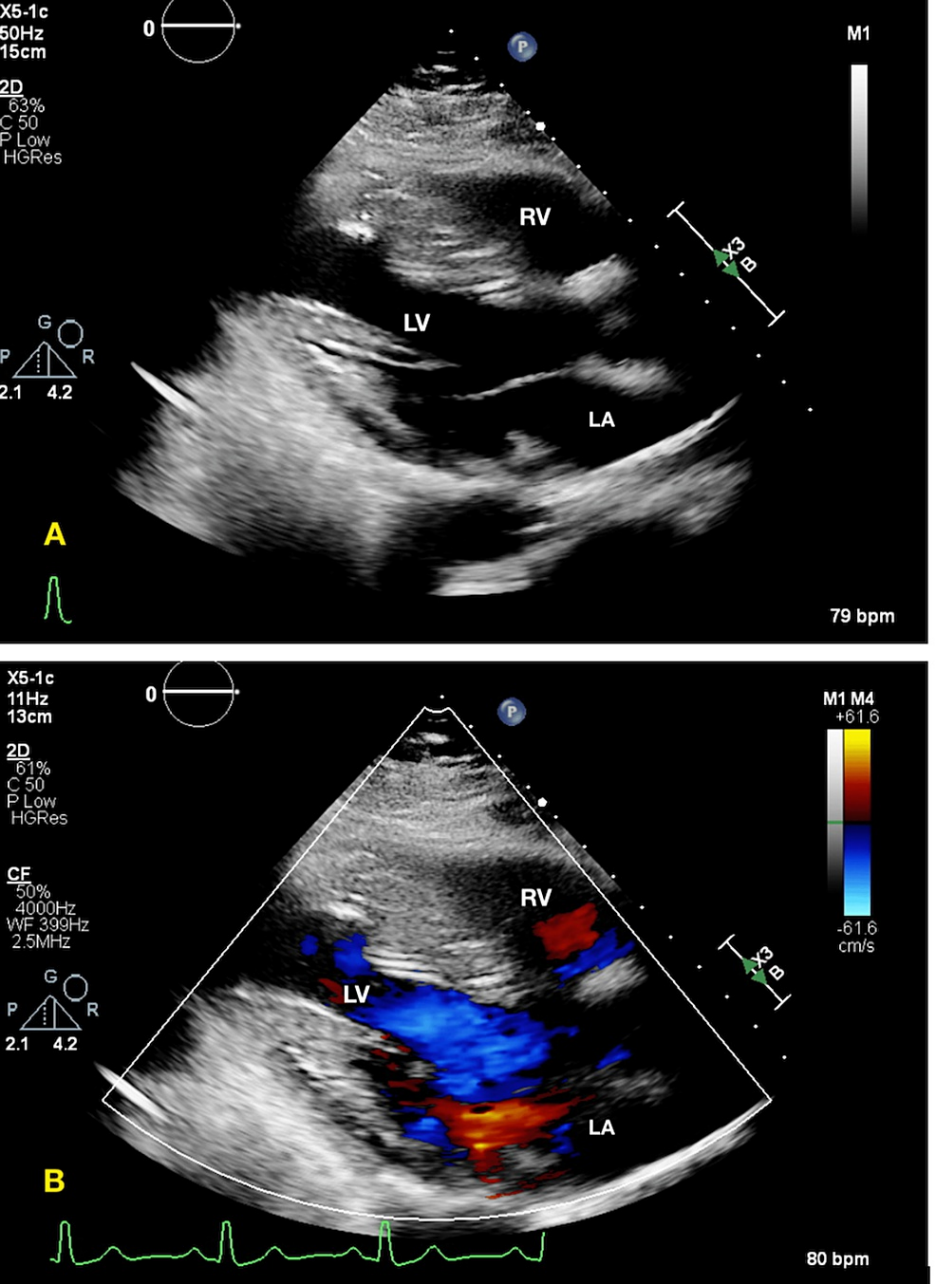
2D Transthoracic Echocardiogram in Parasternal Long Axis demonstrating normal systolic and diastolic function. A: Without doppler flow. B: With doppler flow RV: right ventricle, LA: left atrium, LV: left ventricle

**Figure 7:**
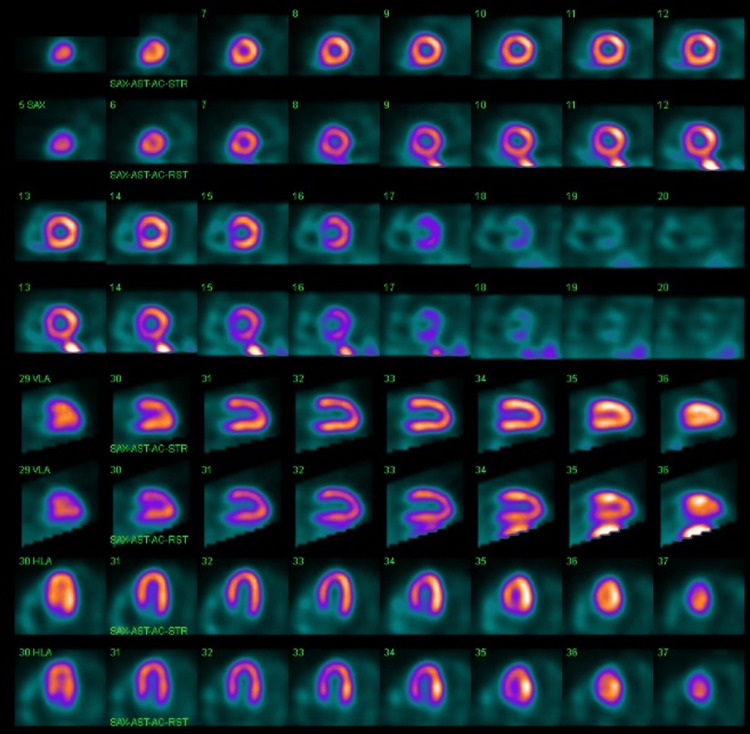
Myocardial perfusion scan without evidence of ischemia.

**Figure 8:**
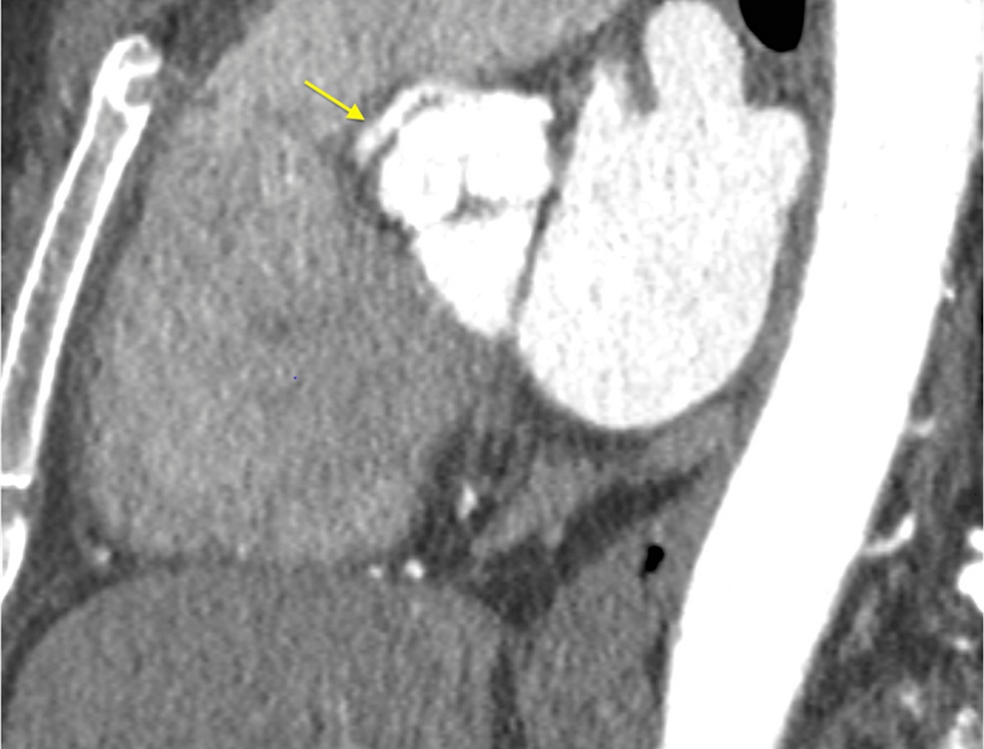
Cardiac computed tomography angiography (CCTA) demonstrating an anomalous RCA originating from above the left sinus of Valsalva with an interarterial course between the aorta and pulmonary artery. The yellow arrow indicates the anomalous RCA with an acute angle takeoff from the aorta. RCA: right coronary artery

TTE showed an ejection fraction of 74%, normal systolic and diastolic function, and no mitral or aortic valvular stenosis (Figure 6). To further investigate the etiology of her anginal symptoms the patient underwent a myocardial perfusion scan. It demonstrated an exaggerated blood pressure response, as her systolic blood pressure reached >190 mmHg during the stress test, but no evidence of abnormal perfusion or ischemia (Figure 7). A 14-day Holter monitoring was without any concerning arrhythmias. CCTA demonstrated no atherosclerotic disease but showed an anomalous RCA originating from above the left sinus of Valsalva taking an interarterial course between the aorta and pulmonary artery; an acute angle takeoff from the aorta was noted, as was a narrowed, slit-like orifice that suggested the presence of an intramural segment (Figure 8). Given the anomalous RCA with high-risk features including interarterial course and intramural segment, in addition to her presenting symptoms, the patient is currently undergoing follow-up for possible surgical repair.

## Discussion

Unlike that seen in typical coronary anatomy where the RCA and the LMCA arise from the right and left coronary cusps of the aorta, respectively, an anomalous coronary artery is a congenital abnormality in which there is a deviation in the origin or course of a coronary artery as it arises from the aorta. Its presence can lead to disastrous complications such as sudden cardiac death (SCD) or less commonly, atypical chest pain, heart failure, syncope or even a complete absence of symptoms [11]. Interestingly, the risk of pathologic significance appears to be correlated with the specific anatomy of the coronary anomaly. In particular, the risk appears elevated when the RCA originates from the opposite sinus with an interarterial course [12,13]. Other high-risk anatomical features include ostial tightness, acute angle takeoff, and the presence of a slit-like orifice, all of which have been shown to demonstrate a consistent association with SCD among the young athlete population [14]. One possible mechanism that links these high-risk features to their complications is compression of the anomalous artery, which can result in ischemia and SCD. The compressive effect may come from the pulmonary artery, although the aorta seems to be the more likely culprit given the high pressures during its vigorous systolic expansion, a phenomenon that can trigger vasospasms or cause the anomalous artery to kink, particularly in the setting of an acute or high takeoff angle [14,15].

To make the diagnosis of an anomalous coronary artery, CCTA is the gold standard. Unlike TTE, which is a primary imaging modality commonly used to investigate cardiac pathologies, CCTA is not limited by shortcomings such as resolution, experience, proficiency, and subjective criteria [11]. Instead, it is considered the gold standard because it offers significant advantages in both spatial and temporal imaging, which can help with identifying high-risk anatomies and subsequently guide management [12,14]. Despite these advantages, however, CCTA is rarely the first imaging modality performed. The initial diagnostic approach of cardiac pathologies usually centers around the clinical presentation, and primary modalities such as EKG or TTE are more commonly employed as part of the initial workup, particularly in patients who present with heart failure or syncope, as was the case for our two patients. In contrast, CCTA usually only becomes a consideration when the initial workup fails to explain, either in part or in full, the patient’s clinical presentation. Because an anomalous coronary artery can present so heterogeneously, the diagnosis is often delayed and can be particularly detrimental in the setting of high-risk coronary anatomy.

Surgical Repair is a class I indication by the 2018 American Heart Association and American College of Cardiology guidelines and the 2020 European Society of Cardiology guidelines for the treatment of patients who present with typical anginal symptoms in addition to high-risk coronary anatomy, including anomalous aortic origin of the coronaries from the right or left coronary sinus [14]. As observed in Case 2, surgery is necessitated to correct the anomaly and mitigate adverse outcomes.

In both cases, CCTA was pursued to more comprehensively evaluate the clinical presentations of heart failure, angina, and syncope in the setting of a negative ischemic workup. In Case 1, TTE and left heart catheterization were unrevealing in the workup for ischemia or occlusive coronary artery disease. In Case 2, TTE, Holter monitoring, and myocardial perfusion scan were similarly negative. The findings on CCTA for both patients were consistent with anomalous coronary artery anatomy and present a likely culprit for their presentations, ultimately guiding management for both patients. Underscoring the importance of maintaining a broad differential diagnosis and utilizing CCTA with a lower threshold when faced with patients who present as seemingly “typical” cases of decompensated congestive heart failure or angina, amongst other clinical presentations. 

## Conclusions

Despite the rarity of coronary anomalies and their heterogeneous presentations, recognizing their significance and role in causing adverse cardiovascular sequelae is crucial, particularly in the setting of non-ischemic disease with no initial identifiable etiologies. A lower threshold for the use of CCTA can help elucidate certain enigmatic presentations, allow for earlier intervention, and improve long-term outcomes.
